# Intracerebroventricular administration of a modified hexosaminidase ameliorates late-stage neurodegeneration in a GM2 mouse model

**DOI:** 10.1371/journal.pone.0315005

**Published:** 2025-01-03

**Authors:** Manuel E. Lopez, Daniel Wendt, Roger Lawrence, Kerui Gong, Hoonsan Ong, Bryan Yip, Joseph Chen, Linley Mangini, Britta Handyside, Alexander Giaramita, Aashish Lamichhane, Melanie Lo, Vishal Agrawal, Jeremy Van Vleet, Amanda Abolhesn, Jessica B. Felix, Isaac Villalpando, Vikas Bhat, Rolando De Angelis, Yuanbin Ru, Ayesha Khan, Sylvia Fong, Terri Christianson, Sherry Bullens, Brett E. Crawford, Stuart Bunting, Mika Aoyagi-Scharber

**Affiliations:** BioMarin Pharmaceutical Inc., Novato, CA, United States of America; The Chinese University of Hong Kong, HONG KONG

## Abstract

The GM2 gangliosidoses, Tay-Sachs disease and Sandhoff disease, are devastating neurodegenerative disorders caused by β-hexosaminidase A (HexA) deficiency. In the Sandhoff disease mouse model, rescue potential was severely reduced when HexA was introduced after disease onset. Here, we assess the effect of recombinant HexA and HexD3, a newly engineered mimetic of HexA optimized for the treatment of Tay-Sachs disease and Sandhoff disease. Enzyme replacement therapy was administered by repeat intracerebroventricular injections in Sandhoff disease model mice with dosing beginning before and after signs of neurodegeneration. As previously observed, HexA effectively increased the lifespan of Sandhoff disease mice by 3.5-fold only when treatment was started before onset of neurodegeneration. In contrast, HexD3 halted motor decline and ameliorated late-stage disease severity even when dosing began late, after neurodegeneration onset. Additionally, HexD3 had advantages over HexA in enzyme stability, distribution potential, and homodimer activity. Overall, our data indicate that advanced therapeutics may widen the treatment window for neurodegenerative disorders.

## Introduction

GM2 gangliosidoses are inherited severe neurodegenerative lysosomal storage disorders of glycosphingolipid catabolism. Mutations in either the *HEXA* or *HEXB* gene cause the GM2 gangliosidoses Tay-Sachs disease (TSD) or Sandhoff disease (SD), respectively. The *HEXA* and *HEXB* genes encode the α and β hexosaminidase subunits, HEXA and HEXB, which heterodimerize (α/β) to form the lysosomal enzyme beta-N-acetyl-hexosaminidase A, or HexA for short. HexA activity is required to prevent the cellular accumulation of glycosaminoglycans, glycoconjugates, and oligosaccharides. HexA deficiency and subsequent accumulation of metabolites results in defective lysosomal function, which severely impacts neuronal health [1–3].

Several issues hinder the use of HexA enzyme replacement as an effective therapeutic option to treat both SD and TSD. HexA is a heterodimer and therefore poses a challenge for manufacturing, as the β subunit homodimer HexB is more stable in physiological conditions compared to either HexA or HexS (α-subunit homodimer) [4, 5]. To circumvent this obstacle, researchers have successfully engineered functional homodimers that share HexA activity [5–7].

Finally, positive preclinical results with HexA gene therapy in SD cat and mouse models and TSD model sheep, including reduction of GM2 accumulation, correction of cellular pathology, and slowing of neurological manifestations, were obtained when treatment was initiated prior to signs of significant neurodegeneration [5, 8–10]. However, enzyme replacement therapy should ideally be able to mitigate disease severity regardless of when treatment is initiated. Even if the enzyme replacement therapy is administered at late stages of disease, the remaining neurons may have the capacity to sustain function and compensate for the prior neurodegeneration if healthy neuronal activity is maintained.

To address these needs, we engineered a recombinant homodimer called HexD3, a HexA derivative, that has equivalent cellular uptake kinetics to that of HexA while retaining the beneficial structure and functions of earlier homodimer constructs [5–7]. Here, the performance of HexD3 is tested head-to-head against HexA at various stages of disease severity and after onset of symptoms in an SD mouse model engineered to have *Hexβ* gene knockout and subsequent loss of the β-subunit; this model has been used extensively as the preclinical mouse model for testing therapies for both SD and TSD [11]. SD model mice show a severe neurodegenerative disease progression culminating in lower limb spasticity, motor deficit, and a shortened lifespan, and reduction of lyso-GM2 ganglioside levels are indicative of enzyme treatment activity. Bis(monoacylglycero)phosphate (BMP), a glycerophospholipid that serves as a lysosomal marker [12], can also be used to determine the health status of the lysosomes in tissue.

In this paper, we demonstrate that exogenous administration of HexD3 enzyme directly to the brain via weekly intracerebroventricular (ICV) injections is well tolerated through the life of the animal and can also correct peripheral disease. BMP provided pharmacodynamic confirmation of the phenotypic correction observed during treatment.

## Materials and methods

### Production and purification

A DNA construct-encoding full-length Hex sequence (**S1 File**) including signal peptide was synthesized (DNA2.0) and inserted into the pXC17.4 (Lonza Biologics, Berkshire, UK) expression vector. The plasmid was transfected into GSKO cells (Lonza) grown in CDCHO media (Invitrogen) with 6mM glutamine at 37°C and 8% CO_2_. High-expressing colonies were selected by 4MU Hex activity assay and grown in suspension.

Purification of Hex enzymes from the harvest culture media was carried out using standard protein purification techniques. The material was adjusted to pH 6.5 throughout. HexA was purified in 2 steps using the anion exchange resin ANX Sepharose FF followed by Capto Phenyl Impres. HexD3 was purified using ANX Sepharose FF followed by a Capto Butyl hydrophobic interaction step. All 3 resins are from Cytiva, formerly GE Healthcare. Final pooled material was buffer exchanged into artificial cerebrospinal fluid (CSF) buffer: 148mM NaCl, 3mM KCl, 0.2mM NaH_2_PO_4_-H_2_O, 0.8mM Na_2_HPO_4_-7H_2_O, pH 7.2 and concentrated to 20 mg/ml. Endotoxin levels were 0.006 endotoxin units (EU)/mg for HexA and 0.004 EU/mg for HexD3.

### Enzyme cellular uptake assay

Enzyme uptake was measured by competition with cation-independent mannose-6-phosphate receptor (CI-MPR) in human SD and TSD fibroblasts (Coriell GM00203 and GM00221). Mannose 6-phosphate (M6P) is used as a competitive inhibitor. Confluent fibroblasts were treated with ß-Hex at 400.0nM final concentration for 24 hours in a 5% atmosphere of CO_2_ at 37°C. After the 24-hour incubation, cells were switched to growth media in the absence of ß-Hex. Cells were then lysed with M-PER lysis buffer at various times over a 14-day sample period. Cell lysates were assayed for ß-Hex activity using a fluorogenic 4MU substrate (EMD Millipore #454428). All enzyme activity assays were an average of 3 or more independent experiments.

### GM2 activity and GM2AP binding assay

Ganglioside monosialylated-2 substrate (GM2; Sigma Cat# G8397), phosphatidylcholine (PC; Sigma Cat# P3556), phosphatidylinositol (PI; Sigma Cat# P2517) and cholesterol (Chol; Sigma Cat# C8667) were used to prepare liposomes at a molar ratio of 10:50:20:20, respectively. Briefly, GM2 (1:1 in toluene:EtOH), PC (2:1 in toluene:EtOH), PI (in chloroform), and Chol (2:1 in chloroform:MeOH) are mixed together in the specified molar ratios, vacuum dried, then resuspended in 1 mL of 1mM Tris buffer, pH 7.4. The GM2 concentration in the final liposomal preparation is 1 mg/mL. Recombinant human GM2 activator protein (GM2AP) containing an N-terminal 6x-histidine tag is prepared in-house at a concentration of 1.26 mg/mL in acidic phosphate-buffered saline (PBS; pH 6.5) containing 10% glycerol. Reaction was performed in 20 μL volume containing 5 μL of the following: assay buffer (0.2M sodium citrate, pH 4.4 containing 1 mg/mL bovine serum albumin [BSA]), GM2 liposomes, hexosaminidase (0.2 mg/mL), and GM2AP (at various concentrations). Reaction was incubated for 1 hour at 37°C then quenched with 50 μL methanol prior to high performance liquid chromatography (HPLC). A 20 μL volume of the quenched supernatant was injected on a C4 RP column (Waters Cat# 186004495). The amount of GM2 and GM3 in the final reaction mix was quantified by mass spectrometry using [M+2H]+2 extracted ions for each ganglioside (m/z of 701 and 600, respectively). Saturation was not observed under these conditions, so specific activity was calculated using the highest concentration of GM2AP with units in nanomoles of GM3 produced/minute/milligram of Hex enzyme.

### MUG/MUGS assays

The enzymatic activity was determined using the fluorescently labeled synthetic substrates 4-methylumbelliferyl-6-sulfo-N-acetyl-β-D-glucosaminide (MUGS; EMD Millipore Cat# 454428) and 4-methylumbelliferyl-N-acetyl-β-D-glucosaminide (MUG; Sigma Cat# M2133) at a final concentration of 1mM in assay buffer (56mM sodium citric acid, 88mM sodium phosphate (dibasic) and 0.5 mg/mL BSA, pH 4.4). Protein samples were added in 1:1 volume with 1mM artificial substrate and incubated for 30 minutes at 37°C. The reaction was quenched by addition of 200 μL of stop buffer (0.5 M Glycine/NaOH, pH 10.7). The reaction signal was read at Ex355nm and Em460nm with 455 nm cut off.

### Animals

All experiments were performed at an Association for Assessment and Accreditation of Laboratory Animal Care-accredited facility and in accordance with Institutional Animal Care and Use Committee-approved guidelines. A colony of SD model mice (B6;129S4-Hexb^tm1Rlp^/J, JAX stock no: 002914) was initially obtained and refreshed yearly from The Jackson Laboratory (JAX). Mice were housed in ventilated cages (1285L Blue Line Tecniplast) and fed Teklad global 18% protein rodent diet (Envigo, irradiated 2018). Heterozygote (Het) females and knockout (KO) males were mated to generate Het and KO siblings. Age-matched wild-type (WT) animals were ordered as B6129SF2/J hybrids (JAX stock no: 101045).

### Animal behavior phenotyping

Nest building was scored as previously described [13, 14]. A nestlet (Ancare) was provided as supplemental cage material with every cage change. When a measurement was desired, the current nest was replaced with a fresh nestlet for the single-housed animal to recreate the nest. After 1 week, nests were photographed and scored on a scale from 0 to 5. An untouched nestlet received a 0, if touched a 1, partially shredded a 2, fully shredded but no structure formed a 3, nest without walls a 4, and a perfectly-formed nest with walls received a 5.

Activity was monitored using Tru Scan activity-monitoring system (Coulbourn Instruments) with some adaptation. The open-field arenas were replaced with home cages containing bedding minus food and water or other supplements (ie, nestlets or huts). The sensor rings were located on the exterior of the cage above the level of the bedding. Animals were allowed to explore the new home-cage environment while the software monitored beam breaks for 10 minutes. Total distance traveled was calculated in centimeters.

Weight measurements of mice were taken weekly after 56 days of age. For survival analysis, endpoint criteria for euthanasia were defined by inactivity during activity monitoring in combination with having reached 20% weight loss from maximum to ensure that chance of recovery or rebound would be low.

### ICV injections

A permanent ICV cannula was implanted into the left lateral ventricle in the mouse at 6 weeks of age. After implantation, mice were single housed in wire-free cages and provided floor access to food pellets and HydroGel cups (ClearH_2_O). After 1 week of recovery and starting as early as 7 to 9 weeks of age, 100 μg bolus of enzyme in 5 μL volume was infused over a period of 5 to 10 minutes. An antihistamine injection of diphenhydramine (Alfa Aesar) at 5 mg/kg was given 10 minutes prior to ICV injections. ICV injections were continued weekly until the end of study or survival endpoint. For non-survival studies, anesthetized animals were euthanized 24 hours after the last ICV injection by cardiac puncture and blood collection followed by perfusion with PBS.

### Tissue processing

After perfusion with buffered saline, brains and other tissues were isolated. The brain was halved into hemispheres and placed in Eppendorf tubes and flash frozen in liquid nitrogen. For tissue processing other than histology, frozen samples were transferred to homogenization tubes preloaded with zirconium oxide beads (cat. REDE-RNA, Next Advance), and 0.6 mL of cold HPLC-grade water (Sigma-Aldrich) was added to each sample. Samples were homogenized with a Bullet Blender (Next Advance) in a cold room (4°C). An aliquot (300 μL) of this homogenate was removed for biomarker mass spectrometry analysis. The remaining tissue homogenate in the lysing tubes was homogenized again after addition of chilled T-Per buffer (Thermo Fisher Scientific) with protease inhibitor cocktail (Thermo Fisher Scientific). The T-Per homogenate samples were centrifuged in a refrigerated table-top centrifuge (Eppendorf) for 15 minutes at max speed (14,000 rpm), and the supernatant was used for activity assays. The protein concentration of all samples was determined using a bicinchoninic acid assay (Thermo Fisher Scientific).

### Ganglioside and BMP biomarker quantification

Gangliosides and BMP were extracted from the water-tissue homogenate described above. Briefly, 50 μL of homogenate (equivalent to 200 μg protein) was extracted with 500 μL 95/5 methanol/glacial acetic acid (v/v) for 2 hours, vortexing briefly every 20 minutes. Samples were spun down for 5 minutes at 5000 rpm, then the supernatant was transferred to a Nanosep Omega 10K centrifugal filter tube (Pall Corporation, Port Washington, NY) and spun at 12,000 rpm for 30 minutes. Samples were analyzed for 3 different gangliosides (GM2, GM3, GA2) and BMP (22:6) content with an Acquity Ultra Performance Liquid Chromatography (UPLC) attached to a Xevo TQ-S micro Triple Quadrupole Mass Spectrometer (Waters Corporation, Milford, MA).

For gangliosides, compounds were separated with an Acquity UPLC Glycan BEH Amide column (1.7 μm, 2.1 x 150 mm; Waters Corporation, Milford, MA). Solvent A was 5mM ammonium acetate in 94.5% acetonitrile, 2.5% methanol, 2.5% water, and 0.5% formic acid. Solvent B was water. The initial solvent composition was 95% A/5% B at a flow rate of 0.4 mL/min. The column was kept at 50°C. The LC elution gradient profile was 95% A/5% B for 2 minutes, ramp to 50% A/50% B over 10 minutes, 50% A/50% B for 10 minutes, then back to 95% A/5% B for 4 minutes. Samples were ionized by electrospray ionization (ESI) in positive ion mode. The capillary voltage was set at 1.0 kV, the desolvation temperature was set at 500°C, and the desolvation gas flow was 1000 L/h. Two precursor-product ion transitions, one for the (d36:1) species, one for the (d38:1) species, were monitored for each of the 3 gangliosides as follows: GM2-1384.7 > 204.1, 1412.7 > 204.1; GA2-1093.6 > 264.3, 1121.6 > 292.3; GM3-1181.5 > 264.3, 1209.6 > 292.3. The sum of the 2 transitions for each ganglioside was used for quantitation. A standard reference curve containing all 3 gangliosides from 100 to 6.25 pg/μL was prepared with standards from Enzo Life Sciences, Inc. (Farmingdale, NY) in 95/5 methanol/glacial acetic acid (v/v).

For BMP phospholipid, compounds were separated with an Acquity UPLC High Strength Silica C18 column (1.7 μm, 2.1 x 150 mm; Waters Corporation, Milford, MA). Solvent A was 5mM ammonium formate in 74% methanol, 25% water, and 1% formic acid. Solvent B was 5mM ammonium formate in 99% methanol and 1% formic acid. The initial solvent composition was 80% A/20% B at a flow rate of 0.1 mL/min. The column was kept at 50°C. The LC elution gradient profile was 80% A/20% B for 1 minute, ramp to 0% A/100% B over 5 minutes, 0% A/100% B for 10 minutes, then back to 80% A/20% B for 4 minutes. Samples were ionized by ESI in negative mode. The capillary voltage was set at 3.5 kV, the desolvation temperature was set at 600°C, and the desolvation gas flow was 1000 L/h. Two precursor-product ion transitions were monitored, 865.5 > 327.3 for BMP (22:6) and 665.4 > 227.2 for BMP (14:0). A standard reference curve containing BMP (14:0) from 100 to 6.25 pg/μL was prepared with a standard from Avanti Polar Lipids, Inc. (Alabaster, AL). Because BMP (22:6) was not commercially available, BMP (22:6) concentrations are reported as equivalents of BMP (14:0).

### Quantitation of the A2G0 metabolite A2G0’ biomarker

Soluble glycan metabolites were extracted and analyzed as previously described [15]. Briefly, free glycans were extracted from homogenized and clarified brain samples and purified on a Hypersep Hypercarbon porous graphitic carbon column (Thermo Fisher Scientific) as per manufacturer’s instructions. Eluted free glycans were dried down in a vacuum concentrator and were subsequently labeled by reductive amination [^12^C_6_]-aniline as previously described [16]. Prior to analysis by glycan reductive isotope labeling with liquid chromatography and mass spectrometry (GRIL-LC/MS) [16], aniline-labeled samples were reconstituted in water.

The A2G0′ hexasaccharide internal standard was generated from its parent compound, the bi-antennary complex N-glycan GlcNAc-Man-(GlcNAc-Man)-Man-GlcNAc-GlcNAc (A2G0, Oxford notation) heptasaccharide (Prozyme, Hayward, CA). The starting compound A2G0 differs from its metabolite A2G0′ by the loss at the reducing end of a single GlcNAc residue. For the standard, this was carried out by digesting 2 nmoles of A2G0 with 800 units of Endo S endoglycosidase (New England Biolabs, Ipswich, MA) in a total volume of 20 μL of 50mM sodium phosphate, pH 7.5 for 18 hours followed by purification on a Sep-PAK C18 SPE cartridge (Waters), per the manufacturer’s instructions, and dried by centrifugal evaporation. The dried A2G0′ (A2G0 prime) glycan standard was then labeled with [^13^C_6_]-aniline [16]. Labeled standard was then dissolved in water for use as an internal standard.

GRIL-LC/MS analysis was performed on an Acquity UPLC system equipped with a Glycan BEH Amide Hydrophilic interaction liquid chromatography column (1.7 μm, 2.1 mm x 150 mm) (Waters, Milford, MA, USA) connected to a LTQ Orbitrap XL mass spectrometer (Thermo Fisher Scientific, Waltham, MA). Solvent A was 100mM ammonium formate in water, pH 4.5, and solvent B was acetonitrile. Initial solvent conditions were 22% A/78% B at a flow rate of 0.2 mL/min. The column temperature was kept at 60°C. Prior to injection, an amount of aniline-labeled sample equivalent to 70 μg of protein was placed in a sample vial along with 10 pmoles of [^13^C_6_]-aniline-labeled A2G0′ internal standard. Glycans were separated using normal phase chromatography with a gradient starting at 22% A/78% B changing to 37% A/63% B over 65 minutes, stepping up to 100% A for 6 minutes, and then ramped back down to 22% A/78% B over 5 minutes and held there for 9 minutes to re-equilibrate the column.

The Orbitrap mass spectrometer was operated in the positive ion mode and the column eluent introduced by electrospray ionization. The capillary temperature was set at 250°C with a spray voltage of 2 kV. The sheath gas flow was set to 58 and the auxiliary gas flow to 9. Full scans were performed at a resolution of 60,000 and a range of 200 to 2250 m/z. A2G0′ metabolite was determined by radiometric comparison of the [^12^C_6_]-aniline-labeled endogenous A2G0′ ion abundance with that of the differentially isotope-labeled internal standard as previously described [17].

### Histology

For lysosomal associated membrane protein-2 (LAMP2) analysis, frozen brain and liver tissues were fixed in formalin and embedded in paraffin. Sagittal sections (7 μm thick) were immunostained with antibody against LAMP2 (Abcam, 25339, 1:500). For antigen retrieval, slides were immersed in Discovery CC1 solution (Ventana, cat: 950–500) for 30 minutes at 95°C. The blocking buffer consisted of a 2% normal donkey serum, 0.1% BSA, and 0.3% Triton solution in 1x tris-buffered saline. Donkey anti-rat Immunoglobulin G (H+L) (Thermo Fisher Scientific, A-21208, 1:500) was used to detect anti-LAMP2 antibodies. Slides were mounted in DAPI Fluoromount (Electron Microscopy Sciences) to visualize cellular nuclei. Signal isolation was conducted via Photoshop (Adobe) and Image J. For both fluorescence, the percent area of the total adjusted signal was utilized. Graphs were generated using GraphPad Prism. Analysis of variance (ANOVA) with Tukey post hoc testing was utilized to analyze the variance between each of the treatment groups. *P* <0.05 was considered to be statistically significant.

For naphthol enzymatic activity analysis, 15 μm sagittal-cut cryosections from the ipsilateral injection-side brain were fixed in a solution containing 2% formaldehyde, 0.2% glutaraldehyde, and 0.01% Nonidet P40 for 15 minutes at room temperature. Sections were then incubated for 3 hours with AS-BI N-acetyl-b-glucosaminide (NAP; Sigma, Cat# N4006-1G) at a concentration of 0.2 mg/ml citrate buffer, pH 4.5. Sections were then transferred to a NAP solution at pH 5.2 with addition of hexazotized pararsaniline (Sigma, Cat# 1804). Sections were then transferred into 1:1 pararsaniline and 4% (w/v) sodium nitrite solution. Sections were immersed for 1 to 2 hours, until red-color deposits were visible. The sections were subsequently immunostained with antibodies against neuronal nuclear protein (NeuN) (Millipore, MAB377, 1:100). Blocking with AffiniPure Fab Fragment Donkey Anti-Mouse IgG (H+L) was utilized to reduce background (715-007-003). Donkey anti-mouse IgG (H+L) highly cross-adsorbed secondary antibody conjugated to Alexa Fluor 488 (Thermo Fisher Scientific, A-21202, 1:500) was used to detect anti-NeuN antibodies. Slides were mounted in DAPI Fluoromount (Electron Microscopy Sciences) to visualize cellular nuclei.

### Mass spectrometry quantification of Hex protein

The amount of Hex protein in the background of SD mouse brain–protein extract was determined using LC-Parallel Reaction Monitoring (PRM)-based targeted MS. Hex peptides (1 μg per sample) were injected into an in-house packed C18 column (ReproSil-Pur120C18AQ, 1.9 μm, 120Å pore size; 75-μm inner diameter, 50-cm length, New Objective) on a Thermo Scientific Easy nLC 1200 nano-LC system. LC-PRM runs for peptide quantification were carried out on a Thermo Scientific Q Exactive mass spectrometer. A run in PRM mode was performed before data acquisition for retention-time calibration using Biognosys’ indexed retention time concept [18]. Signal processing and data analysis were carried out using SpectroDive™ 7.0 (Biognosys, based on mProphet [19]). A Q-value filter of 1% was applied. The absolute quantification was determined by comparing the abundance of the known internal standard peptides with the endogenous peptides. The ratio of the areas under the curve (between the endogenous and reference peptide) was used to determine the absolute levels of Hex enzyme in the samples.

## Results

### HexD3: A modified hexosaminidase homodimer with improvements over HexA

**Fig 1:**
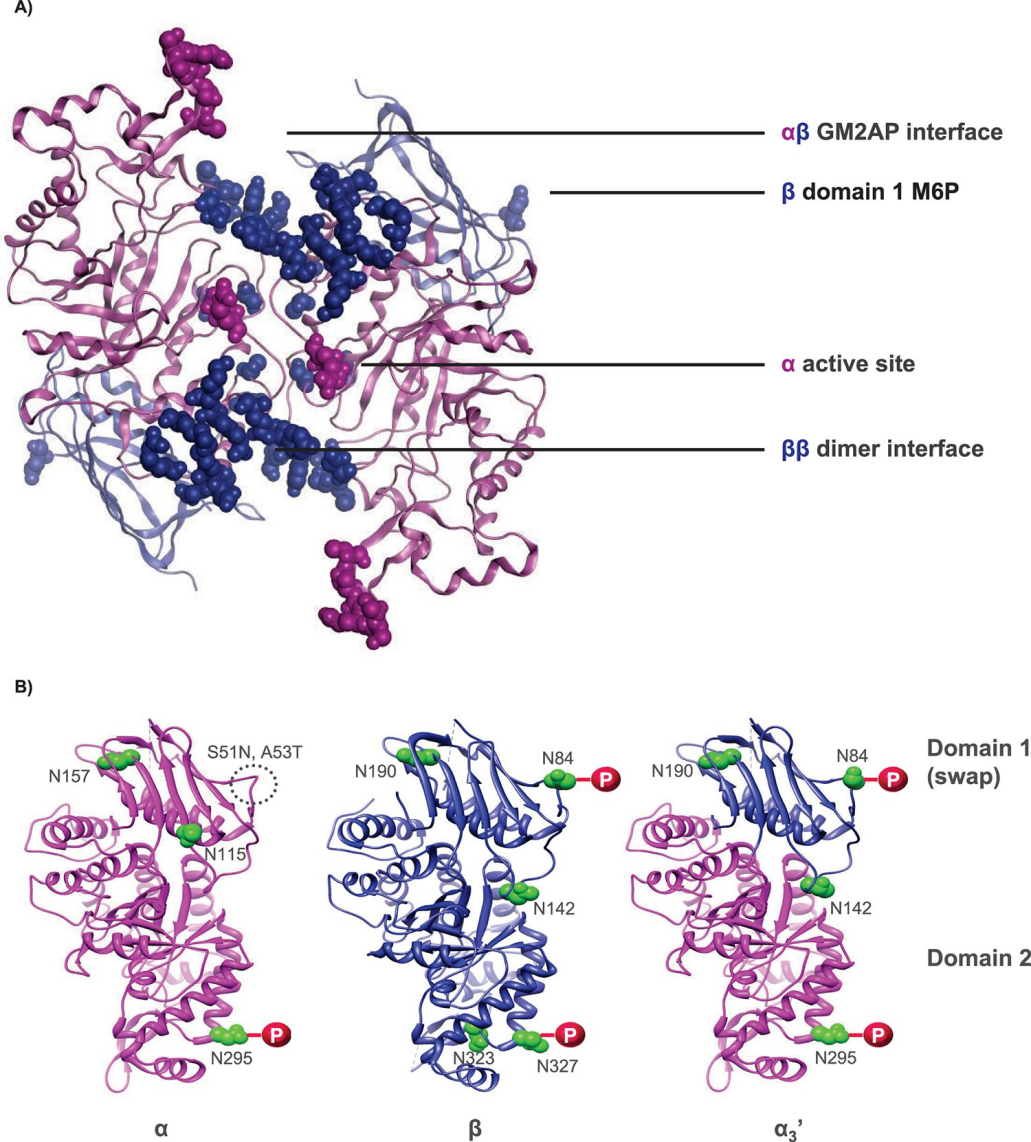
Engineered HexD3 homodimer. A) HexD3 model based on crystal structures of human beta-hexosaminidase A and beta-hexosaminidase isoform B. B) Modification of the HexM α subunit to generate the chimeric α_3_’ subunit contained in HexD3. In panel A, crystal structures of human β-hexosaminidase A (PDB ID: 2GJX) and β-hexosaminidase isoform B (PDB ID: 1NOU) were used to generate the model of HexD3 (https://www.wwpdb.org/). HexD3 retains the GM2AP binding interface of the native α-β heterodimer, the α-subunit enzyme active site, and a β-β homodimer interface of the HexM homodimer [4]. Panel B shows how the parent HexM modified α subunit was further engineered to retain a β domain M6P site. Domain I of the HexM α subunit was replaced with domain I of the native β subunit. The resulting chimeric α_3_’ subunit contains phosphorylated glycans at position N84 compared to the glycosylated, but not phosphorylated, S51N/A53T site in subunit α. Two additional β glycosylation sites, N190/N142, were also included. GM2AP, ganglioside GM2 activator protein; HexA, β-hexosaminidase A; M6P, mannose-6-phosphate; PDB, Protein Data Bank.

HexD3, a newly engineered isoenzyme of HexA, builds upon the structural properties of HexM, a previously reported Hex homodimer (**Fig 1A**) [4]. HexD3 retains the αβ interface for GM2AP binding, the β dimer interface for homodimer stability, as well as the α-subunit active site previously described for HexM [4]. In a unique way, HexD3 contains additional M6P sites to increase receptor-mediated extracellular uptake and delivery to lysosomes [20, 21]. To add M6P sites, the whole domain 1 of the HexM α-subunit scaffold was replaced with the β-subunit domain 1 (**Fig 1B and Table 1**). This modification increased the M6P:homodimer molar ratio to 4:1, which was significantly higher than HexM and native HexA enzymes (2:1 and 3:1, respectively). As a result of the enhanced M6P content, the concentration of enzyme at half-maximal uptake for HexD3, 5.8 ± 1nM MUG activity, is better than HexM (130nM ± 5.4nM) and matches that of HexA (3.3nM ± 0.3nM; **S1 Fig and Table 1**). Prior attempts using single amino acid changes in domain 1 α-subunit (HexD) were unsuccessful in generating additional M6P sites onto HexM (**Table 1, S2 Fig**).

**Table 1:** Comparison of enzymatic and physicochemical properties of Hex variants.

Hex isozyme (name)	HexA	HexM	HexD	HexD3
Gene	HEXA/HEXB	Modified HEXA	Modified HEXA	Modified HEXA
Amino acid substitutions in alpha subunit	**-**	S184K, P209Q, NPVT 228–231 S-LS, P429Q, KD 432–433 RK, I436K, N466A, S491R, LTF 493–495 MDD, E498D, L508V, Q513A, NV 518–519 YA, F521Y, E523N	S184K, P209Q, NPVT 228–231 S-LS, P429Q, KD 432–433 RK, I436K, N466A, S491R, LTF 493–495 MDD, E498D, L508V, Q513A, NV 518–519 YA, F521Y, E523N, **S51N, A53T**	S184K, P209Q, NPVT 228–231 S-LS, P429Q, KD 432–433 RK, I436K, N466A, S491R, LTF 493–495 MDD, E498D, L508V, Q513A, NV 518–519 YA, F521Y, E523N, **alpha 1–165 to beta 1–198**
Subunit composition (dimer)	αβ	α_1_’α_1_’	α_2_’α_2_’	α_3_’α_3_’
M6P-type N-glycan/N-glycan	3/7	2/6	2/8	4/8
M6P contents (mol/mol Hex)	2.5–3	<1	<1	3–3.5
K_uptake_ [nM] MUG activity	3.3 ± 0.3	130 ± 5.4	-	5.8 ± 1.0
Intracellular t_1/2_ MUG activity (day)	14	12	n/a	14
K_m_ GM2AP [μM]	14.5 ± 1.6	19.6 ± 2.5	n/a	13.7 ± 2.4
Specific activity GM2 [nmol GM3/min/mg]	45.8	20.6	n/a	13.6
Specific activity MUG (μmol/min/mg)	66 ± 7	31 ± 3	34	27 ± 2
Specific activity MUGS (μmol/min/mg)	23 ± 3	47 ± 4	52	39 ± 3
T_m_ (°C)	57 ± 0.1	63 ± 0.1	n/a	61 ± 0.2

GM2AP, ganglioside GM2 activator protein; HexA, β-hexosaminidase A; K_m_, Michaelis constant; K_uptake_, concentration of enzyme at half-maximal uptake; M6P, mannose-6-phosphate; MUG, 4-methylumbelliferyl-2-acetamido-2-deoxy-β-d-glucopyranoside; MUGS, 4-methylumbelliferyl-2-acetamido-2-deoxy-β-d-glucopyranoside-6-sulfate; n/a, not assessed; t_1/2_, half-life; T_m_, melting point.

The domain 1 modification in HexD3, in addition to enhanced uptake, also allowed for an improved affinity to GM2AP over HexM, with a K_m_ of 13.7 ± 2.4 μM and 19.6 ± 2.5 μM respectively, compared to 14.5 ± 1.6 μM for HexA (**Table 1**). However, this did not translate into improved GM2-specific activity, with 13.6, 20.6, and 45.8 nmol GM3/min/mg for HexD3, HexM, and HexA, respectively (**Table 1**). Importantly, HexD3 showed activity comparable to HexM on both sulfated and non-sulfated artificial substrates MUGS and MUG. The improved catalytic properties for sulfated substrate MUGS of HexM (47 ± 4 μmol/min/mg) and HexD3 (39 ± 3 μmol/min/mg) compared to native enzyme HexA (23 ± 3 μmol/min/mg) is likely due to the retention of 2 α-subunit active sites in the homodimer. HexA shows better catalytic efficiency on the non-sulfated substrate MUG (66 ± 7 μmol/min/mg) in comparison to HexM (31±3 μmol/min/mg) and HexD3 (27±2 μmol/min/mg). These modified enzymes lack the β subunit active site that favors these neutral substrates [4, 22]. The consequence of this skew in activity for acidic and neutral substrates is unclear. HexD3 was functional in both SD and TSD human fibroblast cells, similar to HexA (**S3 Fig**). Notably, the melting point for both HexD3 (61°C) and HexM (63°C) were more thermostable than HexA (57°C).

### ICV HexA and HexD3 corrected both the brain and periphery

**Fig 2:**
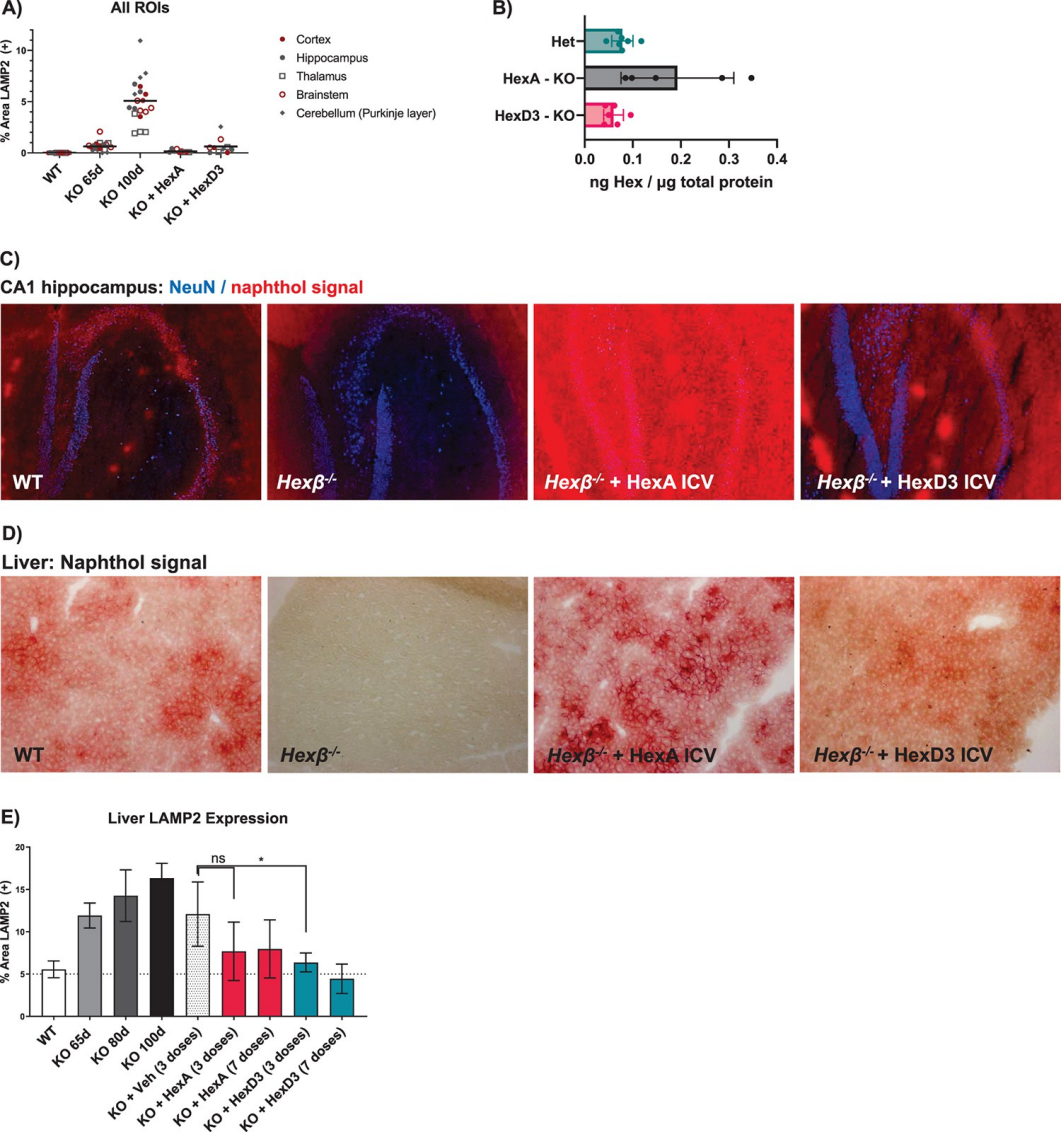
CNS localization and peripheral lysosomal correction following Hex delivery by ICV. A) LAMP2 quantification following doses of HexA or HexD3 in various brain ROIs. B) MS quantitation of concentrations of HexA and D3 in the whole brain hemisphere following ICV dosing. C) Overlay of NeuN and naphthol signal showing localization to neurons in the hippocampus as well as relative concentrations of protein in tissue. D) HexA or HexD3 activity as seen in liver by naphthol signal. E) Liver LAMP2 levels after 3 to 7 doses of Hex enzyme ICV. ns, not significant; * *P* <0.05 using an unpaired t-test. N = 3–5 mice/group. CNS, central nervous system; Het, heterozygote; HexA, β-hexosaminidase A; ICV, intracerebroventricular; KO, knockout; LAMP2, lysosomal associated membrane protein-2; MS, mass spectrometry; NeuN, neuronal nuclear protein; ROI, region of interest; WT, wild type; veh, vehicle.

To understand the biodistribution of the active enzyme in the brain tissue following a bolus ICV injection, LAMP2 staining, a cellular marker of lysosomal disease pathology [23], was evaluated in dissected brain regions. After HexA and HexD3 treatment, LAMP2 staining was lower throughout the brain tissue, even in cerebellum and brain stem regions (**Fig 2A and S4 Fig**). Even after repeated ICV injections (7 doses, once per week), HexA accumulated to a greater extent than HexD3, as detected using MS (**Fig 2B**).

Neurons are the primary targets of Hex-replacement therapy. Therefore, an immunofluorescent neuronal stain, NeuN, was used along with a Hex-reactive naphthol histologic stain to determine whether Hex activity was present in neurons [8, 24]. To confirm co-localization of Hex activity with NeuN, the naphthol signal was converted into a pseudofluorescent signal and overlaid onto the NeuN image (**Fig 2C**). The naphthol stain could be seen throughout the tissue, in and around hippocampal neurons. A strong signal with both HexA and HexD3 was observed in the CA1 hippocampal layer, likely due to its proximity to the ICV injection site.

In addition to treating the lysosomal pathology in the brain, ICV administration also corrected the liver pathology, as expected [25]. Proteins in the cerebrospinal fluid can diffuse into the blood stream and be picked up by peripheral tissue such as the liver. By naphthol staining, HexA showed greater accumulation in the liver than HexD3. HexD3 distribution and level resembled WT most closely (**Fig 2D**). The excess HexA staining concentrated around hepatocytes, possibly in the extracellular space. Compared to vehicle, HexD3 treatment resulted in significantly less LAMP2 staining in the liver (**Fig 2E and S5 Fig**). HexA also decreased LAMP2 staining compared to vehicle, but the difference was not significant.

**Fig 3:**
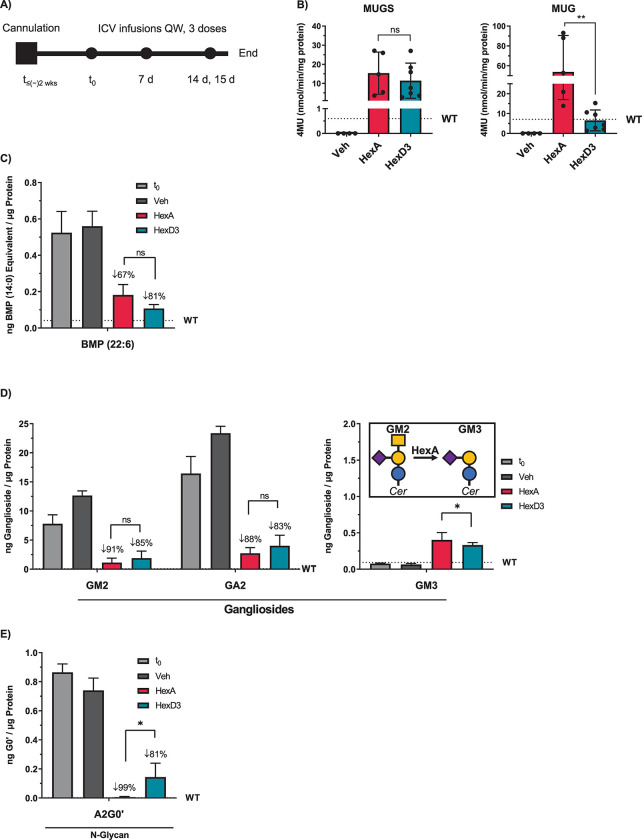
ICV delivery of HexD3 enzyme reduces brain substrates and lysosome BMP similarly to HexA. A) HexA and HexD3 dosing strategy. B) HexA and HexD3 enzyme activity levels found in brain following dosing. C) Brain levels of BMP (22:6) after Hex enzyme dosing. D) Brain levels of GM2, GA2, and GM3 gangliosides after Hex enzyme dosing. E) Brain levels of N-glycan metabolite A2G0’ after Hex enzyme dosing. ns, not significant; * P <0.05; ** *P* <0.01 using a one-way ANOVA. In panel A, brain was harvested for analysis 24 hours after the last dose. In panel B, Sidak’s ANOVA test was used to compare HexA and HexD3. ANOVA, analysis of variance; BMP, bis(monoacylglycero)phosphate; HexA, β-hexosaminidase A; ICV, intracerebroventricular; MUG, 4-methylumbelliferyl-2-acetamido-2-deoxy-β-d-glucopyranoside; MUGS, 4-methylumbelliferyl-2-acetamido-2-deoxy-β-d-glucopyranoside-6-sulfate; t_0_, time zero; t_≤-2wks_, 2 weeks prior to ICV infusion; QW, once weekly; veh, vehicle; WT, wild type.

To investigate the activity in situ of HexD3 compared to HexA, both purified enzymes were delivered to the central nervous system (CNS) by ICV injection into the *Hexβ*^*-/-*^ KO mouse model of SD. Mice were injected at 2 months of age, at asymptomatic stage and prior to CNS deterioration. Three doses, each 1 week apart, were delivered by ICV cannula (**Fig 3A**). Twenty-four hours after the third dose, Hex enzyme activity, the lysosomal biomarker lipid BMP, and Hex enzyme substrates were analyzed from brain homogenates. HexA showed elevated MUG and MUGS activity above WT levels. In comparison, MUG, but not MUGS, activity was significantly lower for HexD3 (**Fig 3B**). The activity still reached WT levels on average. This coincides with the lower specific activity of HexD3 for MUG compared to HexA. Despite this skewed activity for artificial substrate, HexD3 showed an average 81% reduction of lipid BMP, a sign of improved lysosomal health, compared to HexA at only 67% (**Fig 3C**). Furthermore, HexD3 was as effective as HexA at lowering the brain-relative concentrations of gangliosides GM2 and GA2, with 85% and 83% reductions for HexD3, respectively, vs 91% and 88% for HexA, respectively (**Fig 3D**). Inversely, HexA performed better in raising GM3 levels, a product of GM2 metabolism. HexA also lowered levels of N-glycan metabolites known to accumulate in SD [15, 26–28], such as the A2G0 metabolite A2G0′, by 99%, a significantly greater reduction than with HexD3 at 81% (**Fig 3E**).

### HexD3 superior to HexA for treating GM2-disease mice

**Fig 4:**
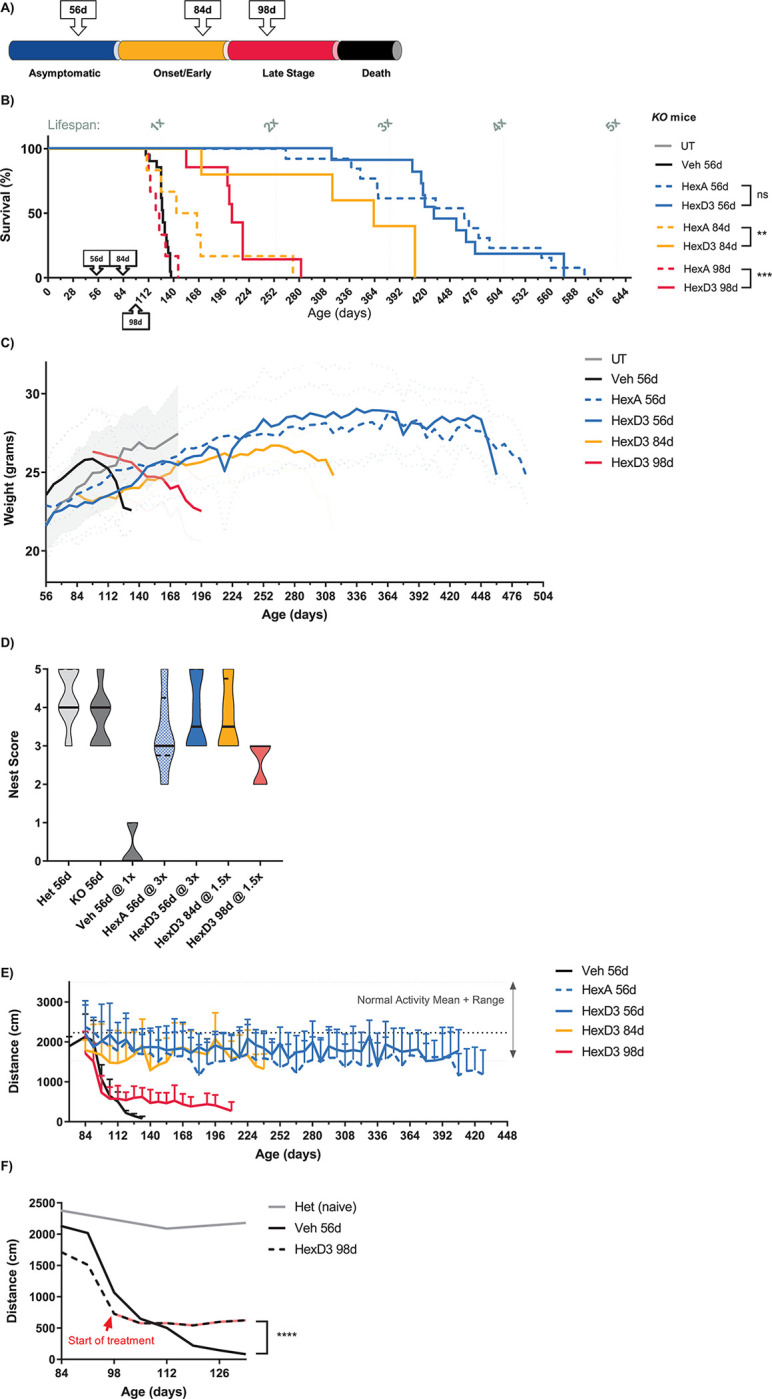
HexD3 ICV can ameliorate late-stage disease progression. A) Schematic showing ages when ICV treatment was started in relation to the targeted stages of disease progression. B) Survival curves demonstrating degree of lifespan benefit compared to untreated disease animals. C) Weight curves and D) nest building evaluating health in animals treated early and late with HexD3. E) Open-field locomotor activity after treatment with HexD3 and HexA beginning at various timepoints. F) Open-field locomotor activity when treatment was started after significant motor loss. ns, not significant; * *P* <0.05; ** *P* <0.01; *** *P* <0.001. N = 4–7 mice/group. For the UT group, n = 60; n ≥6 animals of mixed sex were enrolled per treatment group. In panel A, dotted and solid-colored lines represent HexA and HexD3 enzyme treatments, respectively; blue lines show degree of survival when treatment was started at 56 days of age; yellow lines show the same endpoint at 84 days of age; and red lines at 98 days of age. In panel A, differences between HexD3 and HexA were assessed with a log-rank Mantel-Cox test. In panel C, weight curves were assessed for males only, n ≥3. In panel D, nest building was assessed for mixed-sex groups, n ≥4. In panel E, open-field locomotor activity analysis was assessed in mixed-sex groups, n ≥6. In panel F, differences between vehicle and HexD3 were assessed with an unpaired t-test. For weight and distance, averages with standard deviation were plotted. For nest building, a violin plot was generated with median and quartile marks. All animals are *Hexβ*^*-/-*^ KO mice unless specified as Het, in which case *Hexβ*^*+/-*^ mice were used. Het, heterozygote; HexA, β-hexosaminidase A; ICV, intracerebroventricular; KO, knockout; UT, untreated; veh, vehicle; WT, wild type.

Finally, we evaluated the efficacy of Hex enzyme treatment in *Hexβ*^*-/-*^ KO mice when treatment was initiated at 3 different timepoints corresponding to asymptomatic disease (56 days), onset/early disease (84 days), and late-stage disease (98 days; **Fig 4A**). The median survival age was extended by approximately 3.5-fold the normal lifespan of the disease mice (untreated median, 127 days of age, n = 60) when enzyme delivery (100 μg weekly by ICV) was started during the asymptomatic period at 56 days of age. The survival age of HexA-treated animals (median, 464 days, n = 13) compared to HexD3-treated ones (median, 442.5 days, n = 10) was not significantly different (**Fig 4B**), and their weights were similar throughout the study period (**Fig 4C**). The behavior of these mice was grossly normal aside from some errors in hind limb placement (**S1 and S2 Movies**). Two of the longest-living SD animals (618 days, HexA-treated; 586 days, HexD3-treated) had lifespans close to those of normal mice (600−900 days of age [8, 29]). For comparison, the average lifespans of the B6129SF2/J hybrid mice used as controls and the B6;129S4-Hexb^tm1Rlp^/J mice are around 900 days and 137 days, respectively [11]. The terminal phenotypes of these treated mice varied from sudden death to progressive decline in weight with increasing signs of spasticity, though with less severe effects on gross-locomotor activity with age than in untreated mice.

While HexA and HexD3 treatments displayed comparable effects when initiated at 56 days, surprising differences in their effects emerged when the starting age of treatment was delayed to either 84 or 98 days (**Fig 4B and 4C**). These ages were chosen to mimic a treatment paradigm for early onset and late-stage neurodegenerative pathology. When treatment was started at 84 days of age, the lifespan for HexD3-treated mice (median, 363 days, n = 6) was significantly improved compared with HexA-treated mice (median, 155 days, n = 6). When treatment was started at 98 days of age, HexD3 treatment showed a significant survival benefit (median, 205 days, n = 7) compared to HexA (median, 124 days, n = 6), which was similar to that of vehicle treatment (median, 128 days, n = 20).

Animals treated with HexD3 at 56 and 84 days of age maintained normal (or near normal) nest-building activity (**Fig 4D**), stable weight (**Fig 4C**), and stable motor activity (**Fig 4E**) for the duration of their prolonged lifespans. Animals treated at 98 days of age had a different weight profile from those treated earlier. In this scenario, treatment with HexD3, slowed but did not ultimately change the continued trajectory of weight loss. In contrast, the progressive decline in nest building and motor activity did change, evident from the time of treatment. However, these animals could not recover to normal activity levels even after an extended treatment period. This was most evident with locomotor activity. At the start of treatment, decline in motor activity halted or plateaued for the remainder of the animals’ lifespan (**Fig 4F**). Weight loss was slowed but continued to progress, eventually meeting endpoint criteria.

## Discussion

Our findings indicate that treatment with HexD3, a newly engineered HexA variant, is efficacious in treating the SD mouse model of GM2 gangliosidosis. More importantly, we demonstrate abatement of functional deterioration and death in the SD model when treatment was started late, after significant disease onset. Conversely, HexA treatment failed to achieve the same positive outcome when administered by enzyme replacement therapy, as demonstrated here, or by gene therapy [6]. While positive preclinical results have been obtained with HexA gene therapy administered early in SD cat and mouse models and in TSD sheep [9, 30], including reduction of GM2 accumulation, correction of cellular pathology, and slowing of neurological manifestations, treatment was initiated pre-symptomatically or at early onset, prior to signs of significant neurodegeneration [5, 8–10].

Three key attributes of HexD3 led to improved performance compared to HexA, the natural form of hexosaminidase. First, the thermostability of HexD3 is superior to HexA, and the in vitro CSF half-life of HexD3 is likely longer than HexA, given the HexM parent properties. The improved overall stability of HexD3 is likely due to modified structural attributes such as the addition of the β-subunit glycosylation site N142 in the α_3_’ subunit of HexD3, which is 6 amino acids closer to the N-terminus and replaces the α-subunit counterpart N115. This improved stability is ideal for production of the enzyme, storage, and delivery and likely also benefits its circulation.

HexD3 is a functional homodimer, unlike HexA. Thus far, there have been 2 major contributions toward engineering a homodimer version of HexA: Mod2B, a modified β-subunit that can form a HexA functional homodimer [5], and HexM, a modified α-subunit that can form a HexA functional homodimer [4]. Both Mod2B and HexM take advantage of the strong β-subunit dimer interface found in HexB. HexD3 also incorporates a β-subunit dimer interface, as well as the high substrate activity of HexM, but with improved cellular uptake. When taken up by cells, HexD3 will likely fully retain its homodimer form and activity. In contrast, the heterodimer HexA (comprised of α and β subunits) may recombine into HexB and HexS, thereby reducing overall HexA activity. Therefore, HexD3 not only has an advantage over HexA in terms of thermostability and packing into a therapeutic modality but also as expression of a single biologic. Of note, the reduction of in vivo activity may not be apparent with MUG and naphthol signals in the SD model, since HexB retains activity for these substrates [24], and SD mice have a shunting pathway that would favor HexB activity. Since HexD3 retains the α-subunit scaffold from HexM, it may show overall greater superiority at substrate reduction than HexA in a TSD model.

Finally, HexD3 did not build up in tissues to the extent HexA did. We inferred this from MS and naphthol staining, where intense signal was noted in both the brain, proximal to the site of delivery, and in the liver, in patches of varying intensity for HexA. The signal was stronger in comparison to HexD3 or normal WT tissue. Despite the lower but diffuse amount of tissue distribution of HexD3, there was modest but significantly better improvement in lysosomal correction over HexA. A study using a neuronally expressed HexA transgene showed a similar pattern of HexA localization of activity in the hippocampus [31]. HexA may have been overdosed in our study, and the subsequent accumulation counteracted lysosomal benefit. The maximum concentration of Hex enzymes delivered (100 μg/injection) was based on prior work delivering lysosomal enzymes by ICV injection and prior experience with enzyme replacement therapy in the SD mouse model [32]. We also saw no obvious evidence of tolerability issues from the concentration or frequency of dose used in Het or WT mice (data not shown). More importantly, HexA prolonged survival when treatment was started at an asymptomatic age. Also, at late stage, HexA infusions did not worsen the disease; the lifespan of HexA 98-day-treated animals compared to naïve was not significantly different (*P* = 0.9981). If the levels of HexA were toxic to neurons, the decline in health would be expected to be more severe. While HexA accumulation may not be overtly toxic, the possibility remains that the aggregates can blunt the effectiveness of the enzyme. Thus, despite the native HexA effectiveness at normalizing GM2 and N-glycans in vivo, HexD3 was better at normalizing lysosomal health, as judged by LAMP2 and phospholipid BMP levels.

The benefit seen with HexD3 treatment did not appear to be due to the ICV-delivery modality. If this were the case, HexA would have benefited as well. If indeed HexD3 enzyme replacement therapy is superior to HexA by ICV injection, then it may also work well by gene therapy delivery. In the gene therapy arena, researchers have worked with delivery of 2 halves of an enzyme by co-administering 2 viral vectors, expressing either *HEXA* or *HEXB* genes [8, 33], or a bicistronic construct to ensure expression of both subunits by 1 vector [34]. With engineered forms HexM and HexD3, only a single viral construct is needed. Whether HexD3 is the superior form remains to be tested. In addition, the rescue potential for direct delivery of HexD3 in sheep and cat disease models is necessary to assess the rapidity of enzyme activity and potential pathology correction compared with previous gene therapy results [8–10]. However, ICV delivery of an enzyme may offer certain advantages over gene therapy. We chose ICV delivery over gene therapy to control concentration and quality of enzyme delivered. Expression of enzyme by gene therapy may take time to reach effective levels in the brain and periphery, potentially delaying maximal effect, whereas bolus delivery of HexD3 elicited an immediate correction. The severe decline in open-field locomotor activity plateaued soon after dosing. Rapid rescue of neuronal function may be critical to halt neurodegeneration and recover from loss-of-functions.

We have shown for the first time that modification of late-stage neurodegenerative disease progression in the SD mouse model is feasible. The data points to increasing lysosomal health as the most critical factor for halting CNS deterioration and prolonging the lifespan. HexA and HexD3 both reduced lipid BMP levels in the brain, but the effect size favored HexD3 over HexA (81% vs 67% reduction from untreated, respectively). If the critical therapeutic effect is to reduce the lysosomal burden in both the CNS and periphery, then lipid BMP may become a useful fluid marker to measure lysosomal health in the clinic. Phospholipid BMP accumulates as a secondary storage material in the brains of humans and animals with gangliosidoses and can be detected and quantified in blood and urine [12, 15, 35]. Overall, our results indicate further investigation with HexD3 is warranted.

## Supporting information

S1 File (DOCX)

S1 Fig K_uptake_ of Hex enzymes. Wild-type αß heterodimer (HexA) and modified αα homodimer (HexD3) were shown to be efficiently internalized into SD fibroblasts with an average K_uptake_ ranging from 3nM to 13nM. In contrast, modified αα homodimer HexM exhibited poor uptake efficiency under these experimental conditions (≥120nM). The cell-uptake assay measured ß-Hex uptake by CI-MPR in human SD fibroblasts (Coriell #GM00203). M6P was used as a competitive inhibitor to demonstrate uptake via the CI-MPR receptor. Cells were exposed to the Hex isozymes for 4 hours, washed, lysed, and assayed for enzyme activity using the artificial substrate (MUGS). Each data point represents the average of 3 wells (n = 3). CI-MPR, cation-independent M6P receptor; HexA, β-hexosaminidase A; K_uptake_, concentration of enzyme at half-maximal uptake; M6P, mannose-6-phosphate; MUGS, 4-methylumbelliferyl-6-sulfo-N-acetyl-β-D-glucosaminide; SD, Sandhoff disease; Std., standard; Vmax, maximum reaction rate.
(DOCX)

S2 Fig Hexosaminidase isoenzyme N-glycan profiles. N-glycan profiles from the Hex glycoproteins were generated using PNGase F to cleave asparagine-linked (N-Linked) oligosaccharides from denatured protein. Once cleaved, oligosaccharides were dried and derivatized by reductive amination with the fluorescent dye APTS-M (#725898 from the Carbohydrate Label and Analysis Kit, Beckman-Coulter #477600). The labeled oligosaccharides were applied to a Sephadex G10 spin column (Axygen MSK-100kit) to remove excess dye. The purified oligosaccharides were then separated by capillary electrophoresis. Electrophoresis of samples was performed on the P/ACE MDQ CE (BeckmanCoulter) using a 65-cm N-CAP–coated capillary (#477601) with a 50-μm inner diameter along with the kit-supplied N-CAP buffer (#477603). The laser excitation wavelength for APTS was 488 nm. Man labels indicate an oligomannose-type glycan structure, where Man7, -8, or -9 refers to the repeating mannose monosaccharide units combined to form the glycan structure. APTS-M, 8-aminopyrene-1,3,6-trisulfonic acid; BPM, bis-phosphorylated oligomannose-type glycan structure; HexA, β-hexosaminidase A; MPM, mono-phosphorylated oligomannose-type glycan structure.
(DOCX)

S3 Fig Cellular half-life of Hex isozymes. Cellular stability of HexD3 is on par with known uptake of lysosomal proteins (T_1/2_, 7−14 days). Cells were exposed to the Hex isozymes for 4 hours, washed, lysed, and assayed for enzyme activity using the artificial substrate (MUGS). Each data point represents the average of 3 wells (n = 3). HexA, β-hexosaminidase A; MUG, 4-methylumbelliferyl-2-acetamido-2-deoxy-β-d-glucopyranoside; MUGS, 4-methylumbelliferyl-2-acetamido-2-deoxy-β-d-glucopyranoside-6-sulfate.
(DOCX)

S4 Fig Immunohistochemical images of LAMP2 quantification by treatment group following doses of HexA or HexD3 for the A) cerebral cortex, B) hippocampus, C) thalamus, D) brainstem, and E) cerebellum. Key: DAPI, blue; anti-LAMP2, green. Images were acquired at approximately the same magnification. D, day; DAPI, 4′,6-diamidino-2-phenylindole; HexA, β-hexosaminidase A; HexD3, β-hexosaminidase D3; KO, knockout; LAMP2, lysosomal associated membrane protein-2; WT, wild type.
(DOCX)

S5 Fig Immunohistochemical images for liver LAMP2 levels by treatment group after 3 to 7 doses of Hex enzyme injection by ICV. Every sample was confirmed to have Hex staining via enzyme assay; 65D and 80D had 3 doses, while 100D had 7 doses. D, day; HexA, β-hexosaminidase A; HexD3, β-hexosaminidase D3; ICV, intracerebroventricular; KO, knockout; LAMP2, lysosomal associated membrane protein-2; WT, wild type.
(DOCX)

S1 Movie Home-cage behavior. Mice treated with either HexA or HexD3 from 56 days of age showed grossly normal home-cage behavior and activity. HexA- and HexD3-treatment animals could not be easily distinguished from each other.
(DOCX)

S2 Movie Mice in a cup. Mice were treated with HexD3 from 56 days of age. Mice placed in a confined space with a ledge (such as a shallow Nalgene cup) would attempt to rear to investigate their surroundings. This set-up allows for visual inspection of behavior, foot pad position while rearing, grooming, and ability to move around in a confined space. Treated mice showed normal grooming and rearing activity but with abnormal foot positioning, likely due to some neuronal degeneration. We did not observe worsening of the phenotype with age, as shown in **Fig 4E**.
(DOCX)
