# Supplementary material for: Intracerebroventricular administration of a modified hexosaminidase ameliorates late-stage neurodegeneration in a GM2 mouse model
Source: PLoS One. 2025 Jan 3;20(1):e0315005. doi: 10.1371/journal.pone.0315005 (PMC11698352; doi:10.1371/journal.pone.0315005)
Supplement: S1 File — (DOCX) [file pone.0315005.s001.docx]

# Supplemental material

# Supplemental methods

# HexA and HexD3 sequences

HexA is composed of a heterodimer of α and β subunits. The nucleotide and amino acid sequences of the human α and β subunits are shown below.

## HexA alpha subunit DNA

ATGACTTCCTCCCGCCTTTGGTTCTCCCTCCTGCTTGCCGCTGCCTTCGCCGGACGCGCCACCGCCCTGTGGCCGTGGCCTCAGAACTTCCAGACTAGCGACCAAAGATACGTGCTGTACCCGAACAACTTCCAGTTTCAATACGACGTCAGCAGCGCCGCCCAGCCCGGCTGCTCGGTGCTCGATGAGGCATTCCAGCGGTACCGGGATCTCTTGTTCGGTTCCGGATCATGGCCTCGGCCGTACCTCACTGGAAAGAGGCACACTCTCGAAAAGAACGTCCTGGTGGTGTCCGTGGTCACCCCTGGCTGCAATCAGCTGCCCACCCTGGAATCAGTGGAGAACTACACTTTGACCATCAACGATGACCAATGCCTGCTGCTGTCGGAGACTGTGTGGGGTGCCCTGCGCGGGCTGGAAACCTTTAGCCAACTGGTCTGGAAGTCAGCCGAGGGGACCTTCTTCATTAACAAGACCGAAATCGAGGACTTCCCTCGGTTCCCGCACCGCGGCTTGCTGCTGGATACCTCGCGGCACTATCTGCCACTGTCCTCCATTCTCGATACCCTGGACGTGATGGCCTACAACAAGCTGAACGTGTTCCACTGGCATCTCGTGGACGACCCATCCTTTCCCTACGAGTCCTTCACCTTCCCTGAGTTGATGAGAAAGGGCTCCTACAACCCCGTGACCCATATCTACACGGCTCAGGACGTGAAGGAAGTGATCGAATATGCCCGGCTGAGAGGGATTAGGGTGCTGGCAGAATTTGACACCCCGGGACACACCCTGTCGTGGGGCCCTGGTATCCCGGGCCTGCTGACTCCGTGCTACTCCGGCTCCGAGCCATCCGGAACCTTCGGACCTGTGAATCCCTCCCTGAACAACACTTACGAGTTCATGTCCACCTTCTTCCTGGAAGTGTCGAGCGTGTTCCCCGACTTCTACCTCCACCTCGGTGGCGACGAAGTCGATTTCACTTGCTGGAAGTCTAACCCCGAGATCCAAGATTTCATGCGAAAGAAAGGATTCGGAGAGGACTTTAAGCAGCTGGAGTCCTTCTACATCCAAACCCTGCTGGACATTGTGTCATCGTATGGAAAGGGATACGTGGTGTGGCAGGAAGTGTTTGACAATAAGGTCAAAATTCAGCCCGATACAATCATCCAAGTCTGGCGCGAAGATATCCCCGTGAACTACATGAAAGAACTGGAACTGGTCACGAAGGCTGGATTCAGGGCGCTTCTGAGCGCCCCTTGGTACTTGAACCGGATTAGCTACGGCCCGGACTGGAAGGACTTCTACATCGTCGAACCTCTGGCCTTCGAGGGAACCCCCGAGCAGAAGGCCTTGGTGATCGGCGGCGAAGCCTGTATGTGGGGGGAATACGTGGACAACACCAACCTGGTGCCGCGCCTGTGGCCGAGAGCGGGAGCAGTGGCCGAGCGGCTCTGGTCGAACAAGCTGACTTCCGACCTCACCTTCGCATACGAGAGACTGAGCCACTTCCGCTGCGAACTTCTTCGGCGGGGGGTGCAGGCCCAGCCGCTCAACGTCGGGTTCTGTGAACAGGAGTTCGAGCAGACC

## HexA alpha subunit protein

MTSSRLWFSLLLAAAFAGRATALWPWPQNFQTSDQRYVLYPNNFQFQYDVSSAAQPGCSVLDEAFQRYRDLLFGSGSWPRPYLTGKRHTLEKNVLVVSVVTPGCNQLPTLESVENYTLTINDDQCLLLSETVWGALRGLETFSQLVWKSAEGTFFINKTEIEDFPRFPHRGLLLDTSRHYLPLSSILDTLDVMAYNKLNVFHWHLVDDPSFPYESFTFPELMRKGSYNPVTHIYTAQDVKEVIEYARLRGIRVLAEFDTPGHTLSWGPGIPGLLTPCYSGSEPSGTFGPVNPSLNNTYEFMSTFFLEVSSVFPDFYLHLGGDEVDFTCWKSNPEIQDFMRKKGFGEDFKQLESFYIQTLLDIVSSYGKGYVVWQEVFDNKVKIQPDTIIQVWREDIPVNYMKELELVTKAGFRALLSAPWYLNRISYGPDWKDFYIVEPLAFEGTPEQKALVIGGEACMWGEYVDNTNLVPRLWPRAGAVAERLWSNKLTSDLTFAYERLSHFRCELLRRGVQAQPLNVGFCEQEFEQT

## HexA beta subunit DNA

ATGGAGCTGTGCGGGCTGGGGCTGCCCCGGCCGCCCATGCTGCTGGCGCTGCTGTTGGCGACACTGCTGGCGGCGATGTTGGCGCTGCTGACTCAGGTGGCGCTGGTGGTGCAGGTGGCGGAGGCGGCTCGGGCCCCGAGCGTCTCGGCCAAGCCGGGGCCGGCGCTGTGGCCCCTGCCGCTCTTGGTGAAGATGACCCCGAACCTGCTGCATCTCGCCCCGGAGAACTTCTACATCAGCCACAGCCCCAATTCCACGGCGGGCCCCTCCTGCACCCTGCTGGAGGAAGCGTTTCGACGATATCATGGCTATATTTTTGGTTTCTACAAGTGGCATCATGAACCTGCTGAATTCCAGGCTAAAACCCAGGTTCAGCAACTTCTTGTCTCAATCACCCTTCAGTCAGAGTGTGATGCTTTCCCCAACATATCTTCAGATGAGTCTTATACTTTACTTGTGAAAGAACCAGTGGCTGTCCTTAAGGCCAACAGAGTTTGGGGAGCATTACGAGGTTTAGAGACCTTTAGCCAGTTAGTTTATCAAGATTCTTATGGAACTTTCACCATCAATGAATCCACCATTATTGATTCTCCAAGGTTTTCTCACAGAGGAATTTTGATTGATACATCCAGACATTATCTGCCAGTTAAGATTATTCTTAAAACTCTGGATGCCATGGCTTTTAATAAGTTTAATGTTCTTCACTGGCACATAGTTGATGACCAGTCTTTCCCATATCAGAGCATCACTTTTCCTGAGTTAAGCAATAAAGGAAGCTATTCTTTGTCTCATGTTTATACACCAAATGATGTCCGTATGGTGATTGAATATGCCAGATTACGAGGAATTCGAGTCCTGCCAGAATTTGATACCCCTGGGCATACACTATCTTGGGGAAAAGGTCAGAAAGACCTCCTGACTCCATGTTACAGTAGACAAAACAAGTTGGACTCTTTTGGACCTATAAACCCTACTCTGAATACAACATACGCTTCCTTACTACATTTTTCAAAGAAATTAGTGAGGTGTTTCCAGATCAATTCATTCATTTGGGAGGAGATGAAGTGGAATTTAAATGTTGGGAATCAAATCCAAAAATTCAAGATTTCATGAGGCAAAAAGGCTTTGGCACAGATTTTAAGAAACTAGAATCTTTCTACATTCAAAAGGTTTTGGATATTATTGCAACCATAAACAAGGGATCCATTGTCTGGCAGGAGGTTTTTGATGATAAAGCAAAGCTTGCGCCGGGCACAATAGTTGAAGTATGGAAAGACAGCGCATATCCTGAGGAACTCAGTAGAGTCACAGCATCTGGCTTCCCTGTAATCCTTTCTGCTCCTTGGTACTTAGATTTGATTAGCTATGGACAAGATTGGAGGAAATACTATAAAGTGGAACCTCTTGATTTTGGCGGTACTCAGAAACAGAAACAACTTTTCATTGGTGGAGAAGCTTGTCTATGGGGAGAATATGTGGATGCAACTAACCTCACTCCAAGATTATGGCCTCGGGCAAGTGCTGTTGGTGAGAGACTCTGGAGTTCCAAAGATGTCAGAGATATGGATGACGCCTATGACAGACTGACAAGGCACCGCTGCAGGATGGTCGAACGTGGAATAGCTGCACAACCTCTTTATGCTGGATATTGTAACCATGAGAACATGTAA

## HexA beta subunit

MELCGLGLPRPPMLLALLLATLLAAMLALLTQVALVVQVAEAARAPSVSAKPGPALWPLPLSVKMTPNLLHLAPENFYISHSPNSTAGPSCTLLEEAFRRYHGYIFGFYKWHHEPAEFQAKTQVQQLLVSITLQSECDAFPNISSDESYTLLVKEPVAVLKANRVWGALRGLETFSQLVYQDSYGTFTINESTIIDSPRFSHRGILIDTSRHYLPVKIILKTLDAMAFNKFNVLHWHIVDDQSFPYQSITFPELSNKGSYSLSHVYTPNDVRMVIEYARLRGIRVLPEFDTPGHTLSWGKGQKDLLTPCYSRQNKLDSFGPINPTLNTTYSFLTTFFKEISEVFPDQFIHLGGDEVEFKCWESNPKIQDFMRQKGFGTDFKKLESFYIQKVLDIIATINKGSIVWQEVFDDKAKLAPGTIVEVWKDSAYPEELSRVTASGFPVILSAPWYLDLISYGQDWRKYYKVEPLDFGGTQKQKQLFIGGEACLWGEYVDATNLTPRLWPRASAVGERLWSSKDVRDMDDAYDRLTRHRCRMVERGIAAQPLYAGYCNHENM

## HexD3 DNA

ATGGAACTTTGCGGACTCGGCCTCCCAAGACCACCTATGCTTCTCGCCCTGCTGCTCGCCACCTTGCTCGCGGCTATGCTTGCGCTCCTGACTCAAGTGGCCCTTGTGGTCCAAGTGGCCGAGGCTGCCCGCGCCCCGAGCGTGTCAGCCAAGCCAGGACCGGCCCTGTGGCCGCTGCCTCTGAGCGTGAAGATGACTCCCAATCTCCTGCACCTGGCCCCGGAAAACTTCTACATCTCGCACTCGCCGAACAGCACCGCCGGTCCCTCCTGCACCCTGCTCGAAGAGGCATTCCGGCGGTACCACGGATACATCTTCGGTTTCTATAAGTGGCATCACGAGCCGGCAGAGTTCCAGGCCAAGACTCAGGTCCAGCAGCTGCTCGTGTCCATTACCCTGCAATCGGAGTGCGACGCCTTCCCCAACATCAGCTCAGACGAGTCATACACTTTGCTCGTGAAGGAACCTGTCGCCGTGCTGAAGGCCAACCGCGTGTGGGGTGCCCTGCGCGGGCTGGAAACCTTTAGCCAACTGGTCTACCAAGATTCATACGGGACCTTCACCATTAACGAGTCCACCATCATCGACTCCCCTCGGTTCCCGCACCGCGGCTTGCTGCTGGATACCTCGCGGCACTATCTGCCACTGAAGTCCATTCTCGATACCCTGGACGTGATGGCCTACAACAAGCTGAACGTGTTCCACTGGCATCTCGTGGACGACCAGTCCTTTCCCTACGAGTCCTTCACCTTCCCTGAGTTGATGAGAAAGGGCTCCTACTCCCTCTCCCATATCTACACGGCTCAGGACGTGAAGGAAGTGATCGAATATGCCCGGCTGAGAGGGATTAGGGTGCTGGCAGAATTTGACACCCCGGGACACACCCTGTCGTGGGGCCCTGGTATCCCGGGCCTGCTGACTCCGTGCTACTCCGGCTCCGAGCCATCCGGAACCTTCGGACCTGTGAATCCCTCCCTGAACAACACTTACGAGTTCATGTCCACCTTCTTCCTGGAAGTGTCGAGCGTGTTCCCCGACTTCTACCTCCACCTCGGTGGCGACGAAGTCGATTTCACTTGCTGGAAGTCTAACCCCGAGATCCAAGATTTCATGCGAAAGAAAGGATTCGGAGAGGACTTTAAGCAGCTGGAGTCCTTCTACATCCAAACCCTGCTGGACATTGTGTCATCGTATGGAAAGGGATACGTGGTGTGGCAGGAAGTGTTTGACAATAAGGTCAAAATTCAGCCCGATACAATCATCCAAGTCTGGCGCGAAGATATCCCCGTGAACTACATGAAAGAACTGGAACTGGTCACGAAGGCTGGATTCAGGGCGCTTCTGAGCGCCCCTTGGTACTTGAACCGGATTAGCTACGGCCAGGACTGGAGGAAGTTCTACAAGGTCGAACCTCTGGCCTTCGAGGGAACCCCCGAGCAGAAGGCCTTGGTGATCGGCGGCGAAGCCTGTATGTGGGGGGAATACGTGGACGCGACCAACCTGGTGCCGCGCCTGTGGCCGAGAGCGGGAGCAGTGGCCGAGCGGCTCTGGTCGAACAAGCTGACTAGGGACATGGACGATGCATACGACAGACTGAGCCACTTCCGCTGCGAACTTGTGCGGCGGGGGGTGGCGGCCCAGCCGCTCTACGCGGGGTACTGTAACCAGGAGTTCGAGCAGACC

## HexD3 protein

MELCGLGLPRPPMLLALLLATLLAAMLALLTQVALVVQVAEAARAPSVSAKPGPALWPLPLSVKMTPNLLHLAPENFYISHSPNSTAGPSCTLLEEAFRRYHGYIFGFYKWHHEPAEFQAKTQVQQLLVSITLQSECDAFPNISSDESYTLLVKEPVAVLKANRVWGALRGLETFSQLVYQDSYGTFTINESTIIDSPRFPHRGLLLDTSRHYLPLKSILDTLDVMAYNKLNVFHWHLVDDQSFPYESFTFPELMRKGSYSLSHIYTAQDVKEVIEYARLRGIRVLAEFDTPGHTLSWGPGIPGLLTPCYSGSEPSGTFGPVNPSLNNTYEFMSTFFLEVSSVFPDFYLHLGGDEVDFTCWKSNPEIQDFMRKKGFGEDFKQLESFYIQTLLDIVSSYGKGYVVWQEVFDNKVKIQPDTIIQVWREDIPVNYMKELELVTKAGFRALLSAPWYLNRISYGQDWRKFYKVEPLAFEGTPEQKALVIGGEACMWGEYVDATNLVPRLWPRAGAVAERLWSNKLTRDMDDAYDRLSHFRCELVRRGVAAQPLYAGYCNQEFEQT
