# Supplementary material for: Intracerebroventricular administration of a modified hexosaminidase ameliorates late-stage neurodegeneration in a GM2 mouse model
Source: PLoS One. 2025 Jan 3;20(1):e0315005. doi: 10.1371/journal.pone.0315005 (PMC11698352; doi:10.1371/journal.pone.0315005)
Supplement: S1 Fig — Wild-type αß heterodimer (HexA) and modified αα homodimer (HexD3) were shown to be efficiently internalized into SD fibroblasts with an average Kuptake ranging from 3nM to 13nM. In contrast, modified αα homodimer HexM exhibited poor uptake efficiency under these experimental conditions (≥120nM). The cell-uptake assay measured ß-Hex uptake by CI-MPR in human SD fibroblasts (Coriell #GM00203). M6P was used as a competitive inhibitor to demonstrate uptake via the CI-MPR receptor. Cells were exposed to the Hex isozymes for 4 hours, washed, lysed, and assayed for enzyme activity using the artificial substrate (MUGS). Each data point represents the average of 3 wells (n = 3). CI-MPR, cation-independent M6P receptor; HexA, β-hexosaminidase A; Kuptake, concentration of enzyme at half-maximal uptake; M6P, mannose-6-phosphate; MUGS, 4-methylumbelliferyl-6-sulfo-N-acetyl-β-D-glucosaminide; SD, Sandhoff disease; Std., standard; Vmax, maximum reaction rate. (DOCX) [file pone.0315005.s002.docx]

**Figure S1.** K_uptake_ of Hex enzymes


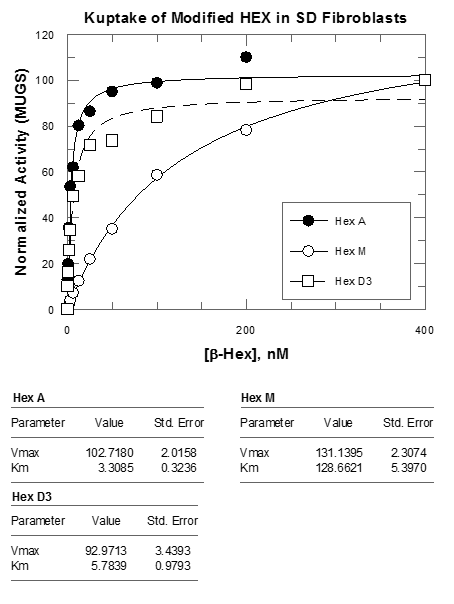


Wild-type αß heterodimer (HexA) and modified αα homodimer (HexD3) were shown to be efficiently internalized into SD fibroblasts with an average K_uptake_ ranging from 3nM to 13nM. In contrast, modified αα homodimer HexM exhibited poor uptake efficiency under these experimental conditions (≥120nM). The cell-uptake assay measured ß-Hex uptake by CI-MPR in human SD fibroblasts (Coriell #GM00203). M6P was used as a competitive inhibitor to demonstrate uptake via the CI-MPR receptor. Cells were exposed to the Hex isozymes for 4 hours, washed, lysed, and assayed for enzyme activity using the artificial substrate (MUGS). Each data point represents the average of 3 wells (n = 3).

CI-MPR, cation-independent M6P receptor; HexA, β-hexosaminidase A; K_uptake_, concentration of enzyme at half-maximal uptake; M6P, mannose-6-phosphate; MUGS, 4-methylumbelliferyl-6-sulfo-N-acetyl-β-D-glucosaminide; SD, Sandhoff disease; Std., standard; Vmax, maximum reaction rate.
