# Supplementary material for: Intracerebroventricular administration of a modified hexosaminidase ameliorates late-stage neurodegeneration in a GM2 mouse model
Source: PLoS One. 2025 Jan 3;20(1):e0315005. doi: 10.1371/journal.pone.0315005 (PMC11698352; doi:10.1371/journal.pone.0315005)
Supplement: S2 Fig — N-glycan profiles from the Hex glycoproteins were generated using PNGase F to cleave asparagine-linked (N-Linked) oligosaccharides from denatured protein. Once cleaved, oligosaccharides were dried and derivatized by reductive amination with the fluorescent dye APTS-M (#725898 from the Carbohydrate Label and Analysis Kit, Beckman-Coulter #477600). The labeled oligosaccharides were applied to a Sephadex G10 spin column (Axygen MSK-100kit) to remove excess dye. The purified oligosaccharides were then separated by capillary electrophoresis. Electrophoresis of samples was performed on the P/ACE MDQ CE (BeckmanCoulter) using a 65-cm N-CAP–coated capillary (#477601) with a 50-μm inner diameter along with the kit-supplied N-CAP buffer (#477603). The laser excitation wavelength for APTS was 488 nm. Man labels indicate an oligomannose-type glycan structure, where Man7, -8, or -9 refers to the repeating mannose monosaccharide units combined to form the glycan structure. APTS-M, 8-aminopyrene-1,3,6-trisulfonic acid; BPM, bis-phosphorylated oligomannose-type glycan structure; HexA, β-hexosaminidase A; MPM, mono-phosphorylated oligomannose-type glycan structure. (DOCX) [file pone.0315005.s003.docx]

**Figure S2.** Hexosaminidase isoenzyme N-glycan profiles

N-glycan profiles from the Hex glycoproteins were generated using PNGase F to cleave asparagine-linked (N-Linked) oligosaccharides from denatured protein. Once cleaved, oligosaccharides were dried and derivatized by reductive amination with the fluorescent dye APTS-M (#725898 from the Carbohydrate Label and Analysis Kit, Beckman-Coulter #477600). The labeled oligosaccharides were applied to a Sephadex G10 spin column (Axygen MSK-100kit) to remove excess dye. The purified oligosaccharides were then separated by capillary electrophoresis. Electrophoresis of samples was performed on the P/ACE MDQ CE (BeckmanCoulter) using a 65-cm N-CAP–coated capillary (#477601) with a 50-μm inner diameter along with the kit-supplied N-CAP buffer (#477603). The laser excitation wavelength for APTS was 488 nm. Man labels indicate an oligomannose-type glycan structure, where Man7, -8, or -9 refers to the repeating mannose monosaccharide units combined to form the glycan structure.

APTS-M, 8-aminopyrene-1,3,6-trisulfonic acid; BPM, bis-phosphorylated oligomannose-type glycan structure; HexA, β-hexosaminidase A; MPM, mono-phosphorylated oligomannose-type glycan structure.
