# Supplementary material for: Intracerebroventricular administration of a modified hexosaminidase ameliorates late-stage neurodegeneration in a GM2 mouse model
Source: PLoS One. 2025 Jan 3;20(1):e0315005. doi: 10.1371/journal.pone.0315005 (PMC11698352; doi:10.1371/journal.pone.0315005)
Supplement: S3 Fig — Cellular stability of HexD3 is on par with known uptake of lysosomal proteins (T1/2, 7−14 days). Cells were exposed to the Hex isozymes for 4 hours, washed, lysed, and assayed for enzyme activity using the artificial substrate (MUGS). Each data point represents the average of 3 wells (n = 3). HexA, β-hexosaminidase A; MUG, 4-methylumbelliferyl-2-acetamido-2-deoxy-β-d-glucopyranoside; MUGS, 4-methylumbelliferyl-2-acetamido-2-deoxy-β-d-glucopyranoside-6-sulfate. (DOCX) [file pone.0315005.s004.docx]

**Figure S3.** Cellular half-life of Hex isozymes


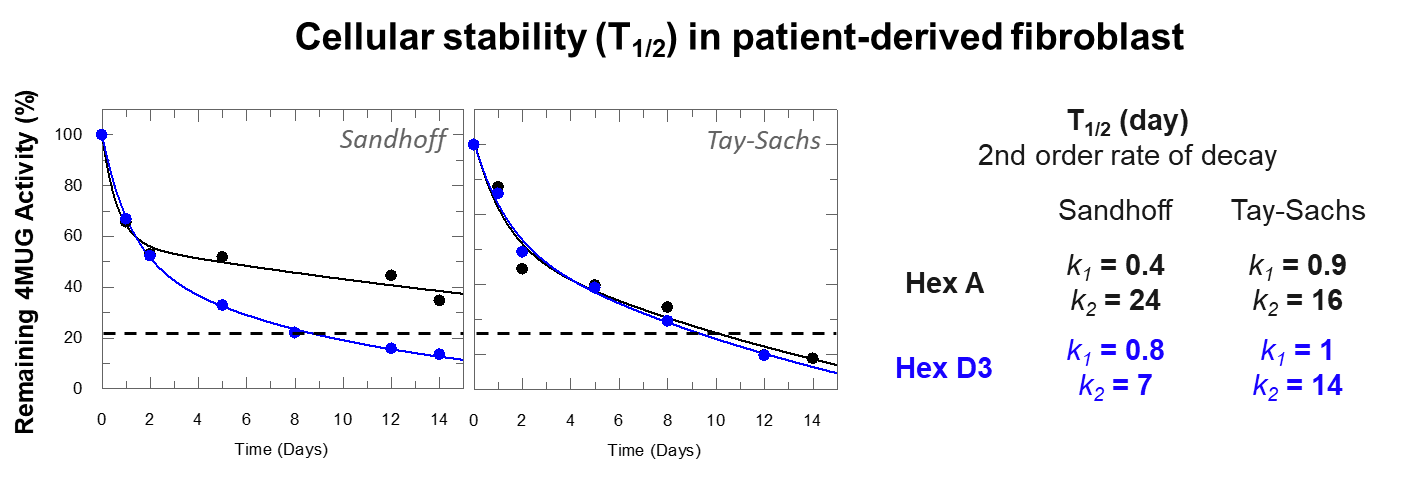


Cellular stability of HexD3 is on par with known uptake of lysosomal proteins (T_1/2_, 7−14 days). Cells were exposed to the Hex isozymes for 4 hours, washed, lysed, and assayed for enzyme activity using the artificial substrate (MUGS). Each data point represents the average of 3 wells (n = 3).

HexA, β-hexosaminidase A; MUG, 4-methylumbelliferyl-2-acetamido-2-deoxy-β-d-glucopyranoside; MUGS, 4-methylumbelliferyl-2-acetamido-2-deoxy-β-d-glucopyranoside-6-sulfate.
