# Supplementary material for: Intracerebroventricular administration of a modified hexosaminidase ameliorates late-stage neurodegeneration in a GM2 mouse model
Source: PLoS One. 2025 Jan 3;20(1):e0315005. doi: 10.1371/journal.pone.0315005 (PMC11698352; doi:10.1371/journal.pone.0315005)
Supplement: S4 Fig — Key: DAPI, blue; anti-LAMP2, green. Images were acquired at approximately the same magnification. D, day; DAPI, 4′,6-diamidino-2-phenylindole; HexA, β-hexosaminidase A; HexD3, β-hexosaminidase D3; KO, knockout; LAMP2, lysosomal associated membrane protein-2; WT, wild type. (DOCX) [file pone.0315005.s005.docx]

**Figure S4.** Immunohistochemical images of LAMP2 quantification by treatment group following doses of HexA or HexD3 for the **A)** cerebral cortex, **B)** hippocampus, **C)** thalamus, **D)** brainstem, and **E)** cerebellum

**A)**

**B)**

**C)**

**D)**

**E)**

Key: DAPI, blue; anti-LAMP2, green. Images were acquired at approximately the same magnification.

D, day; DAPI, 4′,6-diamidino-2-phenylindole; HexA, β-hexosaminidase A; HexD3, β-hexosaminidase D3; KO, knockout; LAMP2, lysosomal associated membrane protein-2; WT, wild type.
