# Supplementary material for: Intracerebroventricular administration of a modified hexosaminidase ameliorates late-stage neurodegeneration in a GM2 mouse model
Source: PLoS One. 2025 Jan 3;20(1):e0315005. doi: 10.1371/journal.pone.0315005 (PMC11698352; doi:10.1371/journal.pone.0315005)
Supplement: S5 Fig — Every sample was confirmed to have Hex staining via enzyme assay; 65D and 80D had 3 doses, while 100D had 7 doses. D, day; HexA, β-hexosaminidase A; HexD3, β-hexosaminidase D3; ICV, intracerebroventricular; KO, knockout; LAMP2, lysosomal associated membrane protein-2; WT, wild type. (DOCX) [file pone.0315005.s006.docx]

**Figure S5.** Immunohistochemical images for liver LAMP2 levels by treatment group after 3 to 7 doses of Hex enzyme injection by ICV

Every sample was confirmed to have Hex staining via enzyme assay; 65D and 80D had 3 doses, while 100D had 7 doses.

D, day; HexA, β-hexosaminidase A; HexD3, β-hexosaminidase D3; ICV, intracerebroventricular; KO, knockout; LAMP2, lysosomal associated membrane protein-2; WT, wild type.
