# Supplementary material for: Intracerebroventricular administration of a modified hexosaminidase ameliorates late-stage neurodegeneration in a GM2 mouse model
Source: PLoS One. 2025 Jan 3;20(1):e0315005. doi: 10.1371/journal.pone.0315005 (PMC11698352; doi:10.1371/journal.pone.0315005)
Supplement: S1 Movie — Mice treated with either HexA or HexD3 from 56 days of age showed grossly normal home-cage behavior and activity. HexA- and HexD3-treatment animals could not be easily distinguished from each other. (DOCX) [file pone.0315005.s007.docx]

**Movie S1.** Home-cage behavior

Mice treated with either HexA or HexD3 from 56 days of age showed grossly normal home-cage behavior and activity. HexA- and HexD3-treatment animals could not be easily distinguished from each other.
