# Supplementary material for: Intracerebroventricular administration of a modified hexosaminidase ameliorates late-stage neurodegeneration in a GM2 mouse model
Source: PLoS One. 2025 Jan 3;20(1):e0315005. doi: 10.1371/journal.pone.0315005 (PMC11698352; doi:10.1371/journal.pone.0315005)
Supplement: S2 Movie — Mice were treated with HexD3 from 56 days of age. Mice placed in a confined space with a ledge (such as a shallow Nalgene cup) would attempt to rear to investigate their surroundings. This set-up allows for visual inspection of behavior, foot pad position while rearing, grooming, and ability to move around in a confined space. Treated mice showed normal grooming and rearing activity but with abnormal foot positioning, likely due to some neuronal degeneration. We did not observe worsening of the phenotype with age, as shown in Fig 4E. (DOCX) [file pone.0315005.s008.docx]

**Movie S2.** Mice in a cup

Mice were treated with HexD3 from 56 days of age. Mice placed in a confined space with a ledge (such as a shallow Nalgene cup) would attempt to rear to investigate their surroundings. This set-up allows for visual inspection of behavior, foot pad position while rearing, grooming, and ability to move around in a confined space. Treated mice showed normal grooming and rearing activity but with abnormal foot positioning, likely due to some neuronal degeneration. We did not observe worsening of the phenotype with age, as shown in **Figure 4E**.
